# Integrating literature and family insights: exploring the needs of families supporting adults with diabetes

**DOI:** 10.3389/fpubh.2024.1473723

**Published:** 2025-01-08

**Authors:** Vânia Lídia Soares, Sara Lemos, Carlos Sequeira, Carminda Soares Morais, Maria do Céu Barbieri-Figueiredo

**Affiliations:** ^1^Institute of Biomedical Sciences Abel Salazar, University of Porto, Porto, Portugal; ^2^Center for Health Technology and Services Research, Faculty of Medicine, University of Porto, Porto, Portugal; ^3^Escola Superior de Enfermagem do Porto, Porto, Portugal; ^4^School of Health, Polytechnic Institute of Viana do Castelo, Viana do Castelo, Portugal; ^5^The Health Sciences Research Unit, Coimbra Nursing School, Coimbra, Portugal; ^6^Centre for Studies and Research in Health, University of Coimbra, Coimbra, Portugal; ^7^Departamento de Enfermeria, University of Huelva, Huelva, Spain

**Keywords:** nursing, family, social support, diabetes mellitus, health impact assessment, nonpharmacological interventions

## Abstract

**Introduction:**

The considerable influence that family members can have on diabetes management is well recognized. Therefore, it is crucial for professionals to acknowledge the impact of the diagnosis on family members. This study aimed to comprehensively identify and understand the needs of family members with an adult diagnosed with diabetes using a two-phased research design.

**Methods:**

Phase 1 was a scoping literature review using databases such as MEDLINE®, CINAHL®, SciELO, and PsycINFO, and gray literature from the Scientific Open Access Repository of Portugal and OpenGrey, focusing on studies from 2017 to 2023, adhering to the Joanna Briggs Institute and PRISMA guidelines. Phase 2 involved a focus group to gather qualitative data on family experiences, which was analyzed using content analysis and following the Consolidated Criteria for Reporting Qualitative Research.

**Results from both phases revealed five themes:**

Communication and emotional expression within families, the impact of diabetes on the family, diabetes-specific knowledge, socio-cultural and environmental influences on diabetes management, and communication with healthcare providers. Integrating these findings highlighted the specific needs of families, suggesting that tailored interventions should be developed to enhance diabetes management support and promote overall family well-being.

## 1 Introduction

Diabetes mellitus (DM) affects 537 million people worldwide, and projections show that around 783 million adults (20–79 years) will be living with this disease (1). Present on global political agendas, DM has a profound impact at various levels, including personal, familial, social, and healthcare systems, due to the severity of its complications and the resources required for its control (1).

Diabetes management and related health outcomes are influenced by various factors, including ethnicity, cultural background, socioeconomic conditions, and access to healthcare. These differences can significantly impact the management of diabetes and related health outcomes.

The growing prevalence of diabetes accentuates the urgent need to develop more effective interventions that go beyond traditional approaches. Therefore, family involvement in diabetes management and interventions has been emphasized in the literature as a determining factor for success, with a positive impact on patient outcomes (2, 3).

However, to promote support for adults with diabetes, effective strategies that holistically address all the factors impacting family members’ health are essential.

The diagnosis of diabetes affects not only individuals but entire families, restructuring daily routines and impacting their emotional and financial well-being (4).

Managing diabetes imposes lifestyle changes such as adhering to a strict diet, regularly monitoring blood glicose levels, and consistently taking medication or insulin therapy (5). These adjustments can be demanding, requiring a collective effort from family members to support the diagnosed person.

Extensive research has been conducted on family involvement in diabetes management and its positive impact on patient outcomes (6, 7). The literature highlights the significant role of family involvement, associating it with improvements in patient outcomes and the maintenance of self-management behaviors (2, 3, 8).

Several family-focused interventions have been developed and assessed in recent years, with their effectiveness demonstrated (9). Most of these interventions aimed to improve family-specific diabetes knowledge and enhance their skills to provide instrumental support to individuals with diabetes (10). However, despite the increase in diabetes-specific knowledge among family members, patients still identify their relationships with family members as critical and often feel that family members can be barriers to changing lifestyle habits (11). These behaviors, described in the literature as non-supportive or sabotaging, may result from researchers and health professionals neglecting the importance of family conflicts, the diversity of relationships among members, and family cultural beliefs (12, 13).

The significant potential that family members have to influence diabetes management is well recognized. Therefore, professionals need to recognize the impact of the diagnosis on family members, particularly those involved in supporting self-management activities or directly performing related tasks and responsibilities (10, 14). Health professionals should recognize and address family emotional needs, how they interact and behave, how they communicate and solve problems, the influence of their health patterns on healthy choices, the cultural impact and beliefs in disease management, and the social-economic and environmental impact of their contexts (14).

Through this comprehensive analysis, professionals and researchers will be able to develop family-tailored interventions that improve diabetes management outcomes and supportive family interaction patterns (10, 15).

This study aims to comprehensively identify and understand the needs of family members and answer the following research questions: What are the specific needs and challenges families face in managing diabetes within their daily routines? How do family members perceive their roles and responsibilities in the care and management of a family member diagnosed with diabetes?

## 2 Methods

A two-phased research design was employed. In phase 1, a scoping review was performed. In phase 2, a one-round focus group survey was conducted with adults with diabetes and family members who support them in disease management.

### 2.1 Phase 1: scoping review

This scoping review was performed according to the Joanna Briggs Institute (JBI) methodology for scoping reviews (16, 17). This Phase 1 aimed to explore and comprehensively identify and understand the needs and perceptions of family members of adults with diabetes described in the literature.

#### 2.1.1 Search strategy

A three-step search was performed by two independent reviewers. An initial search was limited to PubMed and CINAHL, using the search terms “adult with diabetes” OR “diabetic” AND “family needs” OR “family assessment” OR “needs assessment.” This search aimed to identify the indexed and free-text terms most frequently used in articles developed on this topic by analyzing their titles, abstracts, and keywords of the studies. A second search was conducted using indexed search terms and keywords, adapted and individualized to each electronic database, including MEDLINE ®, CINAHL®, SciELO - Scientific Electronic Library Online, and PsycINFO (access via EBSCOhost Web). A third search was developed using gray literature. The gray literature and unpublished studies were mapped using the databases: RCAAP (Scientific Open Access Repository of Portugal), and OpenGrey. The search strategy used for MEDLINE® is provided in Supplementary material 1.

#### 2.1.2 Inclusion criteria

The scoping review search was limited to studies published between 2017 and 2023 to ensure the inclusion of the most recent guidelines and advancements in diabetes care. The inclusion criteria for the scoping review were defined using the PCC (Population, Concept, and Context) mnemonic.

- Population (P): studies conducted with participants that included family members of adults diagnosed with diabetes mellitus, both being 18 years of age or older.
- Concept (C): studies focused on identifying and understanding the specific needs, perceptions, and challenges of family members involved in diabetes management;
- Context (C): studies conducted in any healthcare and community settings, including hospitals, clinics, home care, and community health programs, where family needs in diabetes support are relevant.
- Types of evidence: various studies were included to understand the needs of family members supporting adults with diabetes. Qualitative studies to capture insights and personal experiences and quantitative studies. Mixed-methods studies that integrated both qualitative and quantitative data for a comprehensive understanding. Review articles, including systematic reviews and meta-analyses, synthesized existing research on family involvement in diabetes care.

Studies published in English, Portuguese, Spanish, and French were included based on the reviewers’ level of linguistic proficiency, ensuring greater rigor in the selection of evidence and data extraction.

#### 2.1.3 Data collection

This process was performed by two reviewers. Articles were screened for inclusion using Endnote X9® Software reference management software (Clarivate Analytics, PA, USA), and the duplicate references were identified and removed. Two reviewers independently screened the titles and abstracts. The potentially relevant full-text information was assessed by the same two authors. Disagreements between the two reviewers at any stage of this process were resolved by consensus or through analysis and discussion by a third reviewer. The selection process was guided and presented using the PRISMA Preferred Reporting Items for Systematic Reviews and Meta-Analyses extension for Scoping Reviews -PRISMA-ScR (18).

#### 2.1.4 Data extraction

Two independent reviewers developed the data extraction process for this scoping review. A data extraction tool was developed and tested to ensure comprehensive and accurate collection of relevant information from the selected studies. To ensure accuracy, two independent reviewers conducted data extraction, with discrepancies resolved through discussion or consultation with a third reviewer.

The extracted data included the following information: author(s), year of publication, title, study design and population, and settings. Additional relevant details encompassed outcomes, needs, and perceptions of family members related to family involvement in diabetes management. The synthesis of study information was compiled into a table and presented through a narrative synthesis.

### 2.2 Phase 2: focus group

A qualitative descriptive approach was conducted using focus groups to explore and describe the lived experiences of family members of adults diagnosed with diabetes. We intended to understand how family members perceived the disease and its management without relying on preconceived theories or frameworks (19, 20). The Consolidated Criteria for Reporting Quality Research (COREQ) guidelines were used according to the Enhancing the Quality and Transparency of Health Research (EQUATOR) network (21).

#### 2.2.1 Participants and recruitment

Participants were selected based on specific criteria to ensure relevance to the aim of the study. These included adults diagnosed with diabetes and their family members aged 18 years or older who are involved in diabetes support. Although it is well-recognized that the majority of individuals diagnosed with diabetes are living with type 2 diabetes, no distinction regarding the type of diabetes was made during the recruitment process for the focus group (1). This decision was made to ensure inclusivity and to reflect the diverse experiences and challenges faced by families of individuals with diabetes. The concept of family used for participant selection was “family is who its members say they are” [(22), p.55]. Individuals with cognitive impairments were excluded.

Healthcare professionals at the Family Health Unit made an initial contact by phone to inquire about individuals’ availability to participate in the study. The researcher conducted an initial screening to ensure participants met the inclusion criteria. Efforts were made to ensure a diverse group of participants in terms of age, gender, and socioeconomic status. Participants were grouped, and the focus group was scheduled at a convenient time for them, and reminders were made by phone to confirm attendance.

This focus group was initially conducted to gather participants’ opinions about a proposed intervention being developed for adults with diabetes and their families. However, during this session, several important themes emerged. Given their relevance to understanding the family perspective, these themes will be used in this article to provide a comprehensive understanding of the participants’ perspectives found in the scoping review.

#### 2.2.2 Setting

The study was conducted at a family health unit in Portugal, where the individuals receive primary care services.

#### 2.2.3 Data collection

The focus group took place in May 2024 and comprised adults with diabetes and their family members. After written informed consent, the voice recordings were used for data collection, with the consent of all participants. Voice recordings were intended solely to assist in transcribing the focus group content. The moderator role was assumed by the primary author, a PhD Nursing Science student currently holding a research grant. She has extensive background knowledge on the topic gained through her professional experience in clinical practice and research. This has been her area of investigation for the past 8 years. The moderator always took into account the assumptions inherent to the focus group and ensured a productive discussion by adhering to the principles of focus groups, refraining from giving personal opinions, and steering the conversation toward the study’s objectives (23). Another nurse, also a PhD Nursing Science student with experience in qualitative research, assumed the role of observer and took notes during the session. Data saturation was achieved by identifying the repetition of information. The focus group session took an average of 2 h.

#### 2.2.4 Ethical considerations

The ethics approval for this study was obtained from the Ethics Committee from Alto Minho Local Health (Decree-Law No 97/95 of 10 May, revised by Decree-law No 8072018 of 15 October). All participants who met the inclusion criteria were provided detailed information about the study, including its objectives, procedures, and benefits.

The confidentiality and anonymity of participants’ identities and their responses were ensured by the researchers. Participants were also informed that their participation was voluntary and that they could withdraw from the study at any time without any consequences. All participants were asked to provide written informed consent before participating in the focus groups.

#### 2.2.5 Data analysis

In this study, the content analysis was developed using Mayring’s Qualitative Content Analysis (QCA) (24). This systematic approach allowed us to understand the textual data, prioritizing the creation of a coherent and easily understandable text while respecting grammatical structure and preserving the participant’s original intent. This method provides a clear process that enhances the reliability and validity of the analysis.

We considered the following main phases: (i) formulating a specific research question about family members’ involvement in diabetes management; (ii) pre-analyzing the transcript data and notes and selecting the material relevant to these questions; (iii) developing a coding frame with categories. Finally, a conceptual structure was obtained, composed of the main themes and categories of analysis (24).

#### 2.2.6 Validity and reliability

To ensure the validity and reliability of the focus group data, we followed the guidelines provided by the COREQ (Consolidated Criteria for Reporting Qualitative Research) checklist (21). We ensured the transferability of the results by establishing clear criteria for participant selection and achieving diversity in terms of age, gender, and socioeconomic status. During the focus groups, a trained moderator facilitated the discussions while an observer took detailed notes, ensuring consistent data capture. All sessions were audio-recorded with participants’ consent, and the recordings were transcribed verbatim.

To enhance credibility, we checked the preliminary findings with participants to confirm the accuracy of the interpretations. Inter-coder reliability was ensured by having two independent researchers code the transcripts and resolve discrepancies through discussion. These steps helped maintain the data’s dependability and confirmability, providing a robust foundation for the study’s conclusions. Excerpts from the participants’ statements were included to demonstrate the meanings assigned by family members. Each family member is referred to by the letter “F,” followed by a unique identification number, such as F1.

## 3 Results

### 3.1 Phase 1: scoping review results

**Figure 1:**
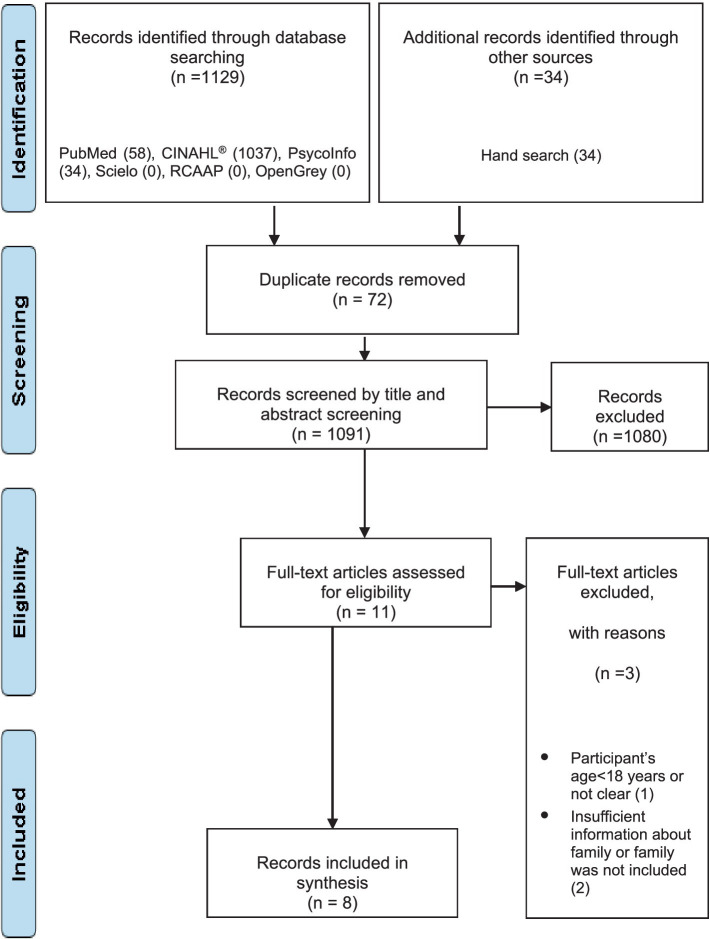
PRISMA flow diagram of study selection.

The search strategy identified 1,163 publications. After excluding 72 duplicates, 1,091 publications were selected for title and abstract analysis. A total of 11 publications were selected for full-text analysis, with 8 studies included in the review. The study selection process is summarized in the PRISMA flow diagram (Figure 1).

All the studies included are written in English. Five of eight were developed in Europe (25–29), two in the United States of America (14, 30), and one in the United Kingdom (30). Regarding the study design, two qualitative studies were included. (26, 30), one described a secondary analysis (31), two were cross-sectional studies (27, 28), one integrative review (25), one was descriptive (14), and one was quantitative (29). The majority of the studies were developed in community settings. More detailed information about studies is described in Supplementary material 2.

**Figure 2:**
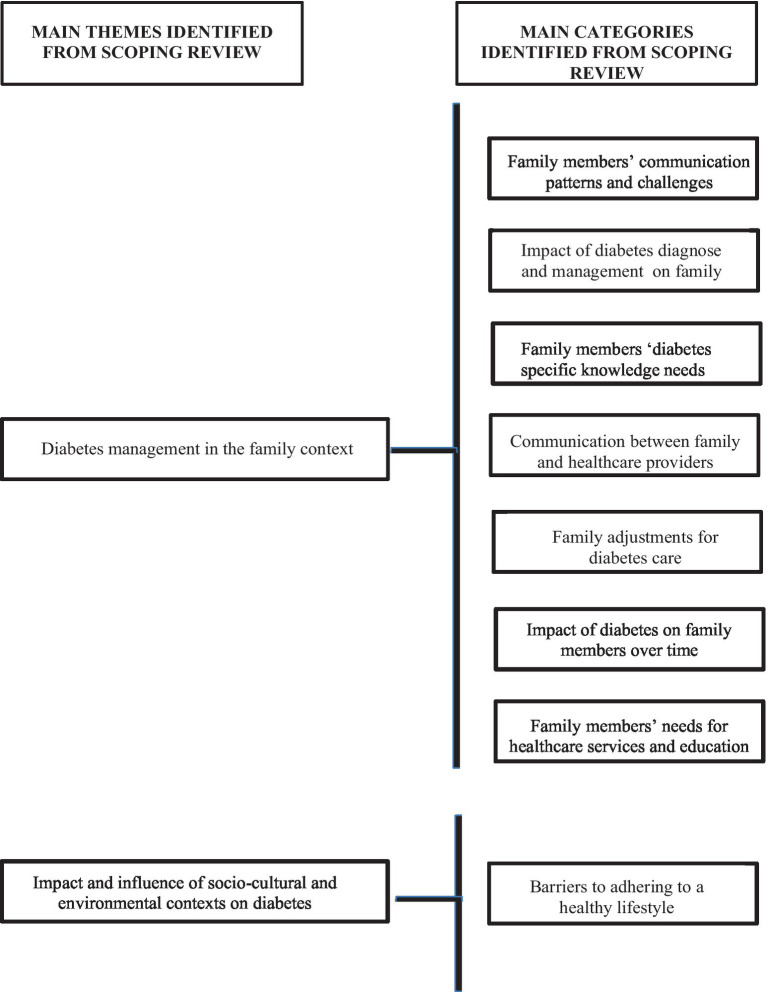
Scoping review overview of the themes and categories.

Based on the guiding questions of this study, the analysis of the studies allowed the identification of needs and perceptions of family members of adults diagnosed with diabetes. The relevant information was gathered and organized into two main themes, each supported by several categories (Figure 2).

The first theme, “Diabetes Management in the Family,” is supported by the following categories:

- Family members’ communication patterns and challenges: disruptive family behaviors were reported, such as arguing about diabetes management, difficulties in communication, gender differences in communication patterns—with men being more authoritative in their speech—challenges in sharing knowledge within the family, inability to influence the diagnosed person, and conflicts with the diagnosed person due to diabetes activities and targets (25, 26, 30).
- Impact of diabetes diagnoses and management activities on family: regarding the diabetes diagnosis and management, family members reported anxiety, stress, anger, distress, frustration, burden regarding diabetes management activities, increased sense of responsibility, lack of confidence, fear, and nervousness facing the diabetes challenges, and feelings of being undervalued in their role in diabetes management by patients (14, 26–31).
- Family members’ diabetes-specific knowledge needs: family members reported a lack of diabetes-specific knowledge, including information about medications, and expressed a desire to engage more frequently with health services to become more involved in diabetes care (14, 26, 30).
- Communication between family and healthcare providers: in this category was reported the lack of time with healthcare providers, confusion about information provided by professionals, and inconsistent information provided in the appointments (14)
- Family adjustments for diabetes care: one study reported family members’ preference to remain uninvolved in diabetes management (25). However, the remaining results showed that most family members expressed significant responsibility for diabetes management activities, its challenges, and targets (14, 26–31).
- Impact of diabetes on family members over time: the diabetes diagnosis and family members’ increased responsibility for diabetes management and patient needs often leads to a decline in family members’ health over time (27, 31). The presence of diabetes complications is highlighted as one of the factors that notably decreases family members’ quality of life (29). The same authors reported several difficulties family members face in maintaining their social connections and support networks, as well as the impact of the disease on the family’s financial conditions.
- Family members’ needs for healthcare services and education: as mentioned above, the lack of knowledge is evident among the relatives of adults with diabetes. Consequently, they also described the need for more regular check-ups to prevent complications and education interventions to improve patient knowledge about complications (29, 30). The same authors described the family members’ need for peer support and psychologist intervention in the disease management process.

The second main theme, “Impact and Influence of Socio-Cultural and Environmental Contexts on Diabetes,” is supported by one category:

- Barriers to adhering to a healthy lifestyle: in this category, family members expressed difficulties in buying healthy and affordable food, societal discrimination against the disease, lack of safe places for physical activity, and inadequate work conditions for managing the diabetes activities by patients (28)

### 3.2 Phase 2: focus group results

The sample comprised 10 participants: five adults diagnosed with diabetes and five family members. All individuals contacted to participate in the study accepted and none dropped out. The reasons for this high participation rate include a strong interest in the topic and the personal relevance of the subject matter to the participants. The adults with diabetes were predominantly male, ranging in age from 58 to 83 years, with a mean age of 71.6 years. Of the five adults diagnosed with diabetes, only one had type 1 diabetes, while the remaining four had type 2 diabetes. Among the participants with type 2 diabetes, all reported using oral antidiabetic medications, with one participant also undergoing combined therapy that included injectable insulin. The duration of diagnosis ranged from 2 to 18 years, with an average of 7.8 years. The family members were predominantly female, aged between 54 and 78 years, with a mean age of 67.4 years. They provided support in diabetes management for an average of 7.8 years, ranging from 2 to 18 years. All participants lived with the person diagnosed with diabetes. The COREQ Checklist details are provided in Supplementary material 3.

**Figure 3:**
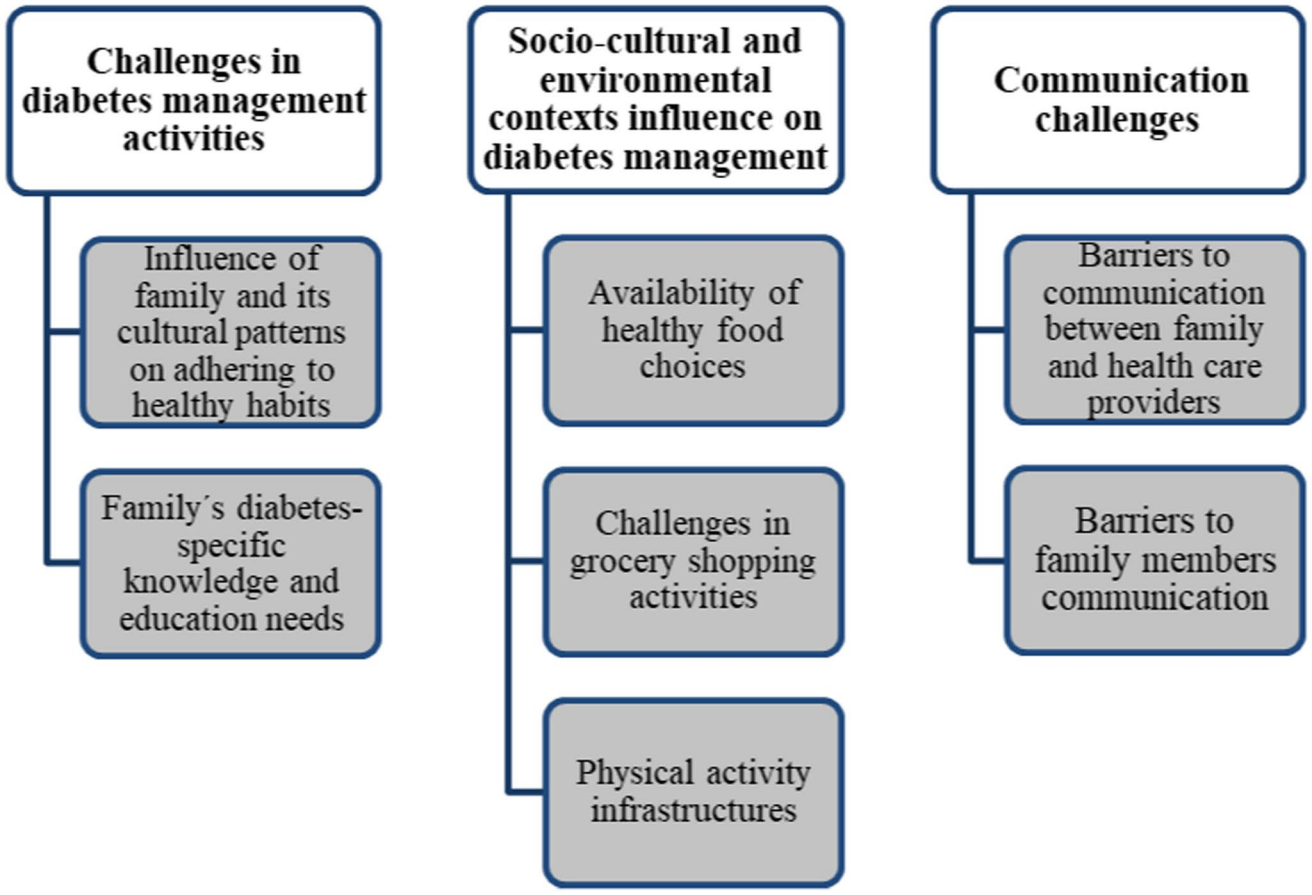
Focus group overview of the themes and categories.

Three themes emerged from data analysis: challenges in diabetes management activities, socio-cultural and environmental contexts influence on diabetes management, and communication challenges. The theme “challenges in diabetes management activities” is supported by two categories: the influence of family and its cultural patterns on adhering to healthy habits and the family’s diabetes-specific knowledge and education needs. The theme “socio-cultural and environmental contexts influence on diabetes management” is supported by three categories: availability of healthy food choices; challenges in grocery shopping activities, and physical activity infrastructures. The third theme, “Communication challenges,” is supported by two categories: barriers to communication between family and healthcare providers and barriers to family members’ communication (Figure 3).

#### 3.2.1 Theme 1: challenges in diabetes management activities

##### 3.2.1.1 Category: influence of family and its cultural patterns on adhering to healthy habits

The participants expressed difficulty adhering to healthy diets. The influence of family and cultural dietary habits on their food choices was evident, and they missed the old foods they ate before their family member was diagnosed with diabetes. They feel they have to eat healthy foods to help their relative who was diagnosed with diabetes, although they need to eat what they want on festive days, independent of patient choices.

“… do you know what it means to eat good food? In this region, we have the best comfort food, but now I cannot eat that... our traditional foods are the best. During Christmas and weddings, I eat a lot of everything and do not care about dietary habits.” F1.

“… good food was in the past, now… we enjoyed eating vegetable soup with beans and bread inside” F2.

“…at home, we do not eat anything like we used to (eat unhealthy food)… now we eat what the doctor recommends, except at Christmas and on birthdays.” F2.

“… it has to be (food choices)... we need to change our diet because we do not want diabetes complications” F1.

Regarding family habits related to regular physical exercise, family members reported difficulty in maintaining regular physical activity habits. Regular physical exercise is not a priority for them, even if their relative with diabetes needs or wants their company or encouragement. They expressed difficulty finding time to exercise due to their busy schedules. Although most are retired, they feel that their daily activities consume much of their time, leaving few opportunities to exercise with the patient. Additionally, they believe that activities such as taking care of their vegetable gardens are sufficient for maintaining their health, ignoring the patient’s needs.

“…more time? I do not have any extra time to spend to walk... I’m retired, but I have a lot to do in my daily life.” F1.

“…I cannot… I have to take care of our vegetable garden, that is a good exercise (…), and I am responsible for picking up my grandchild from school every day at 3:30 PM.” F3.

##### 3.2.1.2 Category: family’s diabetes-specific knowledge and education needs

Family members expressed the need for more information about diabetes management to better support the diagnosed individual, particularly in helping them choose healthy foods. They suggested that interventions would be an effective way to increase their knowledge, specifically group interventions consisting of peers, including the diagnosed individual and their family, as well as professionals. Family members described the need to share knowledge and experiences with peers who also support an adult diagnosed with diabetes.

“…these meetings with people who talk about the same problems we face are important because we get tips and experiences from others regarding diabetes issues.” F1.

“…we need more moments like this to learn about food and medication. We need more tips and advice…” F2.

“… I agree…” F3.

“… I make an effort to read food labels to help my wife choose healthy options… but the list of ingredients is huge... I do not know what those ingredients with strange names are… and which choose…” F2.

#### 3.2.2 Theme 2: socio-cultural and environmental contexts influence on diabetes management

##### 3.2.2.1 Category: availability of healthy food choices

Family members expressed frustration with the limited availability of healthy products in their area of residence. They reported that several local producers have closed their shops, and nowadays, they can only buy more nutritious foods, such as vegetables and fruit, from neighbors (who grow vegetables and other produce), or they have to grow them themselves. They feel that the products available for sale are unhealthy and overly processed.

“…here, the good shops have closed. The bakery with artisanal bread has closed too... now the bread is poorly made and not healthy.” F3.

“...nowadays, everyone has to buy vegetables at the supermarket. I do not like that; it’s not healthy because they use a lot of pesticides. So, I grow my own produce or buy from my neighbors.” F1.

##### 3.2.2.2 Category: challenges in grocery shopping activities

Family members described difficulties in making healthy choices at the grocery store, not only due to a lack of knowledge as previously mentioned, but also because of marketing campaigns. They feel confused by the extensive advertising of ‘light’ and ‘healthier’ products, which are often more prominently displayed. The variety of these products, coupled with the suspicion that some may not be as healthy as claimed, complicates their choices and reflects their concerns about changes in food processing and ingredients. Additionally, they reported several difficulties in interpreting food labels, particularly due to the small print size.

“Every time I go to the grocery store, I see so many yogurts, and I get confused. The doctor said the one I bought was a false ‘light’ yogurt, and others I’ve tried seem to have a lot of something like sugar... it cannot be healthy. Now, I only eat natural yogurt, but I do not like it.” F4.

“In my time, yogurt was made from milk. Nowadays, there are a lot of different ingredients mixed in.” F3.

“I try to read the food labels to help my wife buy healthy food, but I cannot read them. The letters are too small … “F2.

##### 3.2.2.3 Category: physical activity infrastructures

Family members described poor infrastructure conditions for physical activities in their social context, which become more evident during the winter.

“… here we do not have enough places to do exercise in winter, and it is always raining. I practiced swimming, but in the swimming pool it was too cold… and I had a flu… so I gave up… maybe in the summer I return my walkies with my husband or we return” F4.

#### 3.2.3 Theme 3: communication challenges

##### 3.2.3.1 Category: barriers to communication between family and healthcare providers

Family members expressed difficulties in communicating with health professionals due to contradictory information they provided. They felt lost and confused by the differing advice provided by healthcare professionals regarding healthy lifestyles. Furthermore, they find it difficult to question the doctor about issues that bother them regarding support for the diagnosed person. They also mentioned that using medical and technical terms does not help them.

“…the doctor said I have diabetes, but the pharmacist said that with my wife’s blood sugar values, she does not have diabetes.” F3.

“…we received so many different pieces of advice from nurses, doctors, and pharmacists. I do not know what to do. Then there’s the endocrinologist at the hospital... with which values can I say my father is diabetic? The nurse said my father has good glucose values, but the endocrinologist said to continue the rigorous diet.” F5.

“…the doctors start using those technical terms, and I get confused and cannot ask anything.” F2.

##### 3.2.3.2 Category: barriers to family members communication

Family members expressed difficulties in communication within family members. They reported some difficulties in discussing disease management with their relatives without being misunderstood.

“... sometimes it is not easy to talk to her about her food choices.” F2.

“…he is my father, so I have to respect him... but he does not listen to my advice about diabetes.” F3.

### 3.3 Integration of results

**Figure 4:**
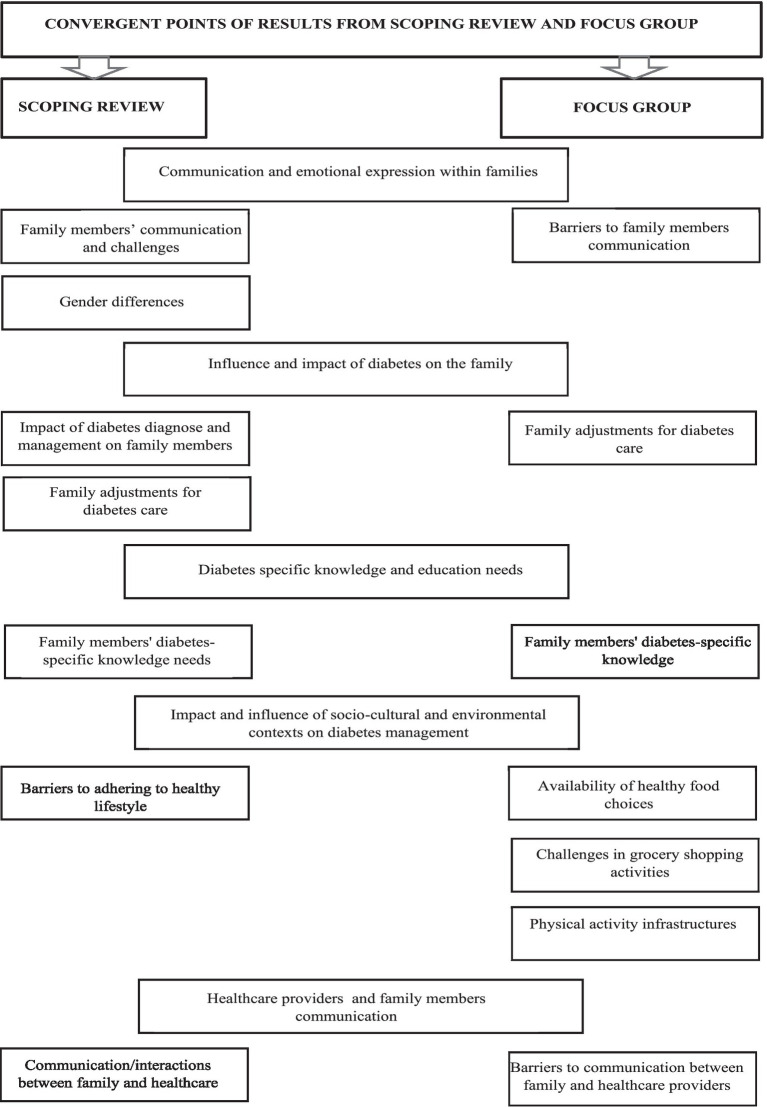
Integrating insights: convergent points and key findings from scoping review and focus group.

The analysis of findings from each methodology allowed us to identify convergent points of the scoping review and focus group results that were gathered in five main themes (Figure 4). Integrating both results was crucial for a comprehensive understanding of the complex dynamics of diabetes management. The five themes provided a multifaceted perspective that combined broad literature findings and, personal experiences from those directly involved. The scoping review offered a systematic overview of existing research, highlighting key areas and gaps in knowledge, while the focus added the perspective of lived experiences, challenges, and needs of families supporting adults with diabetes.

The themes comprised: (i) “Communication and emotional expression within families” is supported by three categories: family members’ communication and challenges, gender differences, barriers to family members communication (ii) “Influence and impact of diabetes management on the family comprised” comprised two categories: impact of diabetes diagnose and management on family members, and family adjustments for diabetes care; (iii) “Diabetes-specific knowledge and education” that is supported by one category identified from scoping review and focus group results: family members’ diabetes-specific knowledge; (vi) “Impact and influence of socio-cultural and environmental contexts on diabetes management” that is supported by four categories: barriers to adhering to healthy lifestyle; availability of healthy food choices; challenges in grocery shopping activities, and challenges in grocery shopping activities (v) “Healthcare providers and family members communication is supported by two categories: communication/ interactions between family and healthcare providers, and barriers to communication between family and healthcare providers (Figure 4).

Regarding the first theme, “Communication and emotional expression within families,” results from both phases identified several difficulties within the family related to communication and emotional expression. Communication and interactions among family members were reported as difficult, often leading to disruptive behaviors such as conflict and arguments over diabetes self-management activities. The differences and difficulties in communication patterns based on gender roles within the family were also emphasized. Family members did not perceive the existence of a supportive environment where they could openly express their emotions and provide mutual support. They reported feelings of confusion about their relative’s health status and felt that the diagnosed person did not recognize their role.

The theme “Influence and Impact of Diabetes Management on the Family” encompassed the category of the “impact of diabetes diagnosis and management on family members” reported in the scoping review results and the “family adjustments for diabetes care” (reported in both phases results).The findings highlighted the significant negative impact that a diabetes diagnosis has not only on the diagnosed adult but also on family members. The responsibilities involved in supporting the diagnosed person were identified as sources of emotional stress, anxiety, and burden for the family. Additionally, family members face various adjustments in their daily lives to support the management of the disease, prevent its progression, and avoid complications, alongside the financial problems imposed by the illness. These combined factors—the emotional and physical strain of caregiving responsibilities, daily life adjustments, and financial pressures—negatively impact family members’ emotional well-being and physical health over time. An important factor to highlight, and that should be the focus of attention for professionals, is the decision of family members not to get involved in diabetes management, or their refusal to adopt the restrictions imposed by disease, even if the diagnosed person expresses a desire for their involvement.

The theme “Diabetes-Specific Knowledge and Education” was reported in the results of both phases. The findings encompass all difficulties related to how family members process and understand health information provided by professionals, specifically focusing on diabetes knowledge and education about disease management. A lack of diabetes-specific knowledge was identified, as well as a need for more education to improve their skills and abilities to support the diagnosed person.

The “Impact and influence of socio-cultural and environmental contexts on diabetes management” was another theme that emerged from results. If the family is influenced by the diagnosis of diabetes, as has been described throughout this study, the importance of the impact of the different contexts in which the family is embedded is undeniable. All external circumstances related to the availability of healthy food and the existing infrastructure for physical activity can act as facilitators or barriers to the family’s adherence to a healthy lifestyle. Thus, in this study, several difficulties and barriers were reported by family members in their daily lives, including a lack of healthy food choices, difficulties in selecting appropriate items in grocery stores, and inadequate conditions for practicing physical activity. These factors significantly negatively influence the daily diabetes management activities and impact the overall health and well-being of family members.

The theme “Healthcare providers and family members communication” was reported in the results of both study phases. In this category, family members reported a lack of time with health professionals, confusion due to the information provided, and contradictory advice received during different consultations. They felt lost and confused by the varying recommendations given by health professionals regarding healthy lifestyles. Additionally, they experienced difficulty in addressing concerns with the doctor about supporting the diagnosed individual. They also mentioned that the use of medical and technical terms was unhelpful.

## 4 Discussion

Adults’ diabetes self-management is a crucial aspect of disease management and typically occurs within a family and social environment (32). Therefore, the various forms of support that families provide to adults with diabetes (e.g., instrumental, social, and emotional) significantly impact diabetes outcomes (10).

Family members’ involvement in diabetes management and interventions is complex and should involve more than increased family member-specific diabetes knowledge as a strategy to improve diabetes control and patient diabetes self-care (33).

It is very valuable that family members can perceive and effectively play their role in successful disease management. This involves understanding how to provide emotional, instrumental, and practical support to their relative with diabetes (34, 35). Equally important is their ability to recognize and use other sources of social support, such as community resources, support groups, and healthcare professionals, when necessary (36).

The family’s role in reinforcing and maintaining the patient’s behavior change is crucial. Literature shows that ignoring this factor could explain the failure of some diabetes family interventions (37).

Family support and involvement in diabetes management should be identified and understood through a comprehensive approach that allows a structured analysis of family dynamics, developmental stages, and functional roles within the context of diabetes (10). Thus, this study, which assessed families using a theoretical model combined with both methodologies (scoping review and focus group), provided a comprehensive framework for understanding the real challenges and needs experienced by families (22).

Family interventions are mostly focused on the outcomes at the individual level and on the overall well-being of the patient (10). Although some studies have assessed the impact of diabetes on family members, the overall impact of diabetes on families is often overlooked (7).

The findings of this study demonstrated the complexity of the construct under analysis and the factors that can affect it. Therefore, both study phase results demonstrated the negative impact on the well-being of family members of emotional dynamics within the adult diagnosis. Family members expressed anxiety, burden, and lower quality of life associated with the higher responsibilities regarding diabetes management (14, 26, 28–31). Family members also reported feelings of exclusion by healthcare providers or the patient, indicating their important role in disease management was not recognized (25, 28, 30). Additionally, their inputs were often overlooked, and they felt unable to influence the diagnosed person.

Family members described these emotional responses, coupled with the low level of diabetes-specific knowledge and education, in both study phases. Furthermore, family members described this as significantly impacting their ability to assist effectively with disease management, as described by other authors (14, 26, 30, 34, 37).

Findings suggested that the emotional dynamics within the family, such as the emotional, psychological, and practical burdens placed on family members, can increase when the diagnosed person’s individual needs conflict with those of the family (28, 29, 37).

Different authors have recognized and demonstrated the importance of family relationships and communication patterns (4, 34, 37, 38). Therefore, positive communication within family can encourage the adult with diabetes on lifestyle modification (4). Conversely, the presence of disruptive family behaviors described have impacted in the well-being of family as described above. These findings align with other studies’ results that highlighted the importance of family relationship quality and communication patterns in improving the well-being of all family members (3, 37, 39). Additionally, these authors noted that interpersonal conflicts and miscommunication can arise when family support is perceived as paternalism and criticism, depending on how the support is communicated and provided. Another important topic that emerged in this study is gender difference in communication based on family roles. Women’s communication patterns were more assertive, while men tended to give orders (25). This can be explained by the traditional responsibilities of women within the family, which are closely linked to their role in taking care of others, especially when a relative is living with diabetes (38).

Developing a supportive role in assisting the adult with diabetes, family member will be exposed to a series of changes in their daily life, starting with practical adjustments required to maintain family well-being. These include integrating changes into their routines and habits. Over time, the impact on family members extends beyond their emotional and psychological well-being, affecting their physical health and overall well-being (27–29, 31).

One topic highlighted by this study is that the presence of someone (family member/friend) in the daily life of an adult with diabetes is not necessarily synonymous with being a source of support. A family member, feeling affected in some way by the diabetes diagnosis, may decide to remain uninvolved in diabetes management and its associated restrictions (25).

The family economic factor also emerged in the findings of this study (29). These authors describe the burden caused by financial problems related to diabetes diagnosis. The literature describes similar results, emphasizing the associations between a lack of family resources and the negative impact on maintaining healthy behaviors and diabetes management outcomes (40, 41). Other authors reported similar findings, which note that individuals with diabetes and low income are less likely to take their medication due to financial concerns with other necessities, such as paying bills (10).

The environmental factors, whose importance in the prevention and control of noncommunicable diseases has been highlighted in international literature, were also described in the results of this study (5, 42). The environmental barriers described included the lack of accessible places to buy healthy food and safe spaces for physical activity, which significantly reduces the ability of families to maintain healthy behaviors (28, 29). The scarcity of healthy food options implies an effort from families to provide nutritious meals for effectiveness. Similarly, the absence of convenient and safe locations for physical activity limits opportunities for regular exercise, which is vital for controlling blood sugar levels and overall health (5, 42).

The societal discrimination against individuals with diabetes was described in one study included in the scoping review (28). This can exacerbate the challenges faced by families with an adult with diabetes and can reduce the community support available for them (5). In addition, the discrimination can lead to feelings of isolation and frustration, which are usually extended to the work environment, as described in the literature (43). Other studies also described these results (40, 41). These challenges experienced by family members could explain the needs expressed by them in this study’s results, which included the presence of a psychologist in diabetes management, as well as the need for more education about the disease and its complications through peer support (29, 30).

The lack of social support is described as a factor that is associated with the worst diabetes outcomes. Therefore, since sources of support are not always available or accessible on a day-to-day basis and may not provide sufficient support to meet the needs of families in managing diabetes, the importance of training and peer support becomes crucial. Peers emerge as individuals who experience the same daily challenges and experiences, and can offer additional social and emotional support that is helpful in dealing with daily diabetes challenges (34, 44, 45).

Considering all the factors described above, addressing the emotional needs of families and supporting their involvement in diabetes-related activities is essential to enhance family support and achieve better outcomes for both patients and their families. Beyond acquiring knowledge and perceived ability to support their relatives, family members may benefit more directly from being actively involved in diabetes management and interventions. This involvement should reduce their stress related to the disease and its complications while also improving their own health behaviors.

### 4.1 Limitations

Regarding the limitations of this study, the following should be noted: the guiding script for the focus group was not checked in a pretest, and the transcripts from the focus group were not returned to participants for comments and/or corrections. Another limitation is the reliance on data from only one focus group, which may not fully capture the diversity of experiences and perspectives among all family members involved in diabetes management. Additionally, the decision to include only studies published from 2017 onwards in the scoping review, while intended to reflect recent advances in clinical guidelines, family-centered interventions, and technological developments, may have excluded valuable insights from earlier studies. The impact of diabetes on family members is a long-standing issue that transcends temporal and technological boundaries, and excluding older studies might have limited the comprehensiveness of the review. Future research could address these limitations by incorporating a broader temporal scope in literature reviews and ensuring a more robust methodology in the qualitative components of the study. Another limitation of this study is the absence of a distinction between type 1 and type 2 diabetes among the focus group participants. This may have impacted the breadth of insights collected and constrained the exploration of challenges unique to specific types of diabetes, particularly those associated with type 1 diabetes and insulin therapy management.

### 4.2 Recommendations

This study comprised two phases, providing a comprehensive understanding of the needs and challenges faced by family members of an adult with diabetes. Healthcare providers should create a supportive environment that enhances the well-being of both adults with diabetes and their family members, leading to more effective diabetes management and improved health outcomes. The development and implementation of tailored diabetes education programs for both the adults with diabetes and their relatives should be based on the holistic assessment of specific needs and strengths within the family, encompassing physical, emotional, social, and economic factors.

By addressing the multifaceted aspects of family dynamics and support, these interventions can foster better communication, stronger emotional support, and more effective management strategies, ultimately improving the quality of life and health for all involved. Future studies should consider conducting separate focus groups for families managing type 1 diabetes and type 2 diabetes. This approach would enable researchers to comprehensively explore the unique needs, challenges, and dynamics associated with each type of diabetes. By doing so, tailored recommendations and interventions could be developed to better support families in managing the specific demands of T1D, such as insulin therapy and glycemic variability, as well as the long-term lifestyle modifications often emphasized in T2D management.

## 5 Conclusion

In this study, the careful planning and systematic integration of the scoping review results and focus group findings allowed us to create a comprehensive article that offers valuable insights drawn from both literature and lived experiences. This approach provided a deeper understanding of family dynamics from different perspectives and highlighted the specific needs of family members, thereby enhancing the robustness of our conclusions.

It is crucial for professionals to recognize the significant role families play in managing the disease of an adult with diabetes, along with understanding the family as a unit of care, and the specificities of diabetes management. This recognition should lead to the active involvement of family members in care planning and decision-making processes.

Based on this knowledge, practitioners should develop more effective and tailored interventions to improve support for diabetes management while simultaneously promoting the overall well-being of the entire family.

## Data Availability

The original contributions presented in the study are included in the article/Supplementary material, further inquiries can be directed to the corresponding author.
