# Supplementary material for: Integrating literature and family insights: exploring the needs of families supporting adults with diabetes
Source: Front Public Health. 2025 Jan 8;12:1473723. doi: 10.3389/fpubh.2024.1473723 (PMC11751045; doi:10.3389/fpubh.2024.1473723)
Supplement: Supplementary file 1 [file Table_1.DOCX]

| **Search** | **Query** | **Results** |
| --- | --- | --- |
| **S1** | diabetes OR diabet* OR “diabetes mellitus” | 1,005,634 |
| **S2** | family OR famil* OR peers OR friend* OR neighbour* OR “fellow patient*” OR colleague* OR “internet contact*” OR “pen friend*” | 2,178,200 |
| **S3** | “family needs” OR “family assessment” OR “needs assessment” | 40,675 |
| **S4** | education intervention OR family nursing | 1,177,846 |
| **S5** | S1 AND S2 AND S3 AND S4 | 192 |
|  | Limiters: English; Portuguese; Spanish; French, from 2017 | 58 |
| **Total** |  | 58 |

Supplementary file 1. Search strategy used for Medline
