# Supplementary material for: Integrating literature and family insights: exploring the needs of families supporting adults with diabetes
Source: Front Public Health. 2025 Jan 8;12:1473723. doi: 10.3389/fpubh.2024.1473723 (PMC11751045; doi:10.3389/fpubh.2024.1473723)
Supplement: Supplementary file 2 [file Table_2.DOCX]

Supplementary material File 2. Details of studies included in the scoping review

| **Author(s)** | **Year of publication** | **Title** | **Study design** | **Population** | **Setting** | **Country** |
| --- | --- | --- | --- | --- | --- | --- |
| Bennich et al. | 2017 | Supportive and non-supportive interactions in families with a type 2 diabetes patient: an integrative review | Integrative review | Spouse or partner represented the family. | Not clear | Sweden |
| Grabowski et al. | 2017 | Involvement of family members in life with type 2 diabetes: Six interconnected problem domains of significance for family health identity and healthcare authenticity | Qualitative study | Family members | Not clear | Denmark |
| Lee et al. | 2017 | Family Members’ Experiences Supporting Adults with Chronic Illness: A National Survey | Descriptive study | Family and friends | Patient’ homes (in and out) | United States of America |
| Costa & Pereira | 2018 | Predictors and moderators of quality of life in caregivers of amputee patients by type 2 diabetes | Cross-sectional study | Patient and caregivers | Community | Portugal |
| Wakefield, & Vaughan-Sarrazin, | 2018 | Strain and Satisfaction in  Caregivers of Veterans with Type 2 Diabetes | Secondary analysis | Patient and caregivers (family/friends) | Community | United States of America |
| Berry et al. | 2019 | Educational and Psychological Aspects Exploring the perceptions of emotional distress among couples living with Type 2 diabetes and among diabetes healthcare providers, and consideration of support needs | Qualitative study | Patients and partners | Community | United Kingdom |
| Nascimento do Ó et al. | 2022 | Interpersonal Relationships in Diabetes: Views and Experience of People with Diabetes, Informal Carers, and Healthcare Professionals in Portugal | Cross-sectional study | Patients, family members and health professionals | Community | Portugal |
| Kolari´c et al. | 2023 | The Quality of Life of Caregivers of People with Type 2 Diabetes Estimated Using the WHOQOL-BREF Questionnaire | Quantitative study | Patients and caregivers | Health care service | Croatia |
